# Exploring the *in vitro* and *in vivo* antileishmanial potential of Marizomib against *Leishmania amazonensis* and *Leishmania infantum*

**DOI:** 10.1128/aac.00286-25

**Published:** 2025-07-21

**Authors:** Patrícia de Almeida Machado, Pollyanna Stephanie Gomes, Juliana da Trindade Granato, Ari Sérgio de Oliveira Lemos, Bruno Vicente, Victor do Valle Midlej, Elaine Soares Coimbra, Herbert Leonel de Matos Guedes

**Affiliations:** 1 Departamento de Patologia, Faculdade de Medicina, Universidade Federal Fluminense 162382, Niterói, Brazil; 2 Laboratório de Imunologia Clínica, Instituto Oswaldo Cruz, Fundacao Oswaldo Cruz-Fiocruz 37903, Rio de Janeiro, Brazil; 3 Laboratório de Imunobiotecnologia, Instituto de Microbiologia Paulo de Góes, Universidade Federal do Rio de Janeiro 28125, Rio de Janeiro, Brazil; 4 Núcleo de Pesquisas em Parasitologia (NUPEP), Instituto de Ciências Biológicas, Universidade Federal de Juiz de Fora 28113, Juiz de Fora, Brazil; 5 Laboratório de Biologia Estrutural, Instituto Oswaldo Cruz, Fundacao Oswaldo Cruz-Fiocruz 37903, Rio de Janeiro, Brazil; The Children's Hospital of Philadelphia, Philadelphia, Pennsylvania, USA

**Keywords:** leishmaniasis, *Leishmania amazonensis*, *Leishmania infantum*, proteasome inibitors, Marizomib

## Abstract

Current treatment available for leishmaniasis is fraught with numerous problems, so the search for new treatment alternatives for leishmaniasis is urgent and necessary. The proteasome has been selected as a promising target. Marizomib is a proteasome inhibitor and has shown antitumor effects, with ongoing clinical tests. In this work, we aimed to evaluate the *in vitro* and *in vivo* effects of Marizomib on *Leishmania amazonensis* and *Leishmania infantum*. Interestingly, Marizomib was not effective against promastigote forms of *L. amazonensis* and *L. infantum in vitro* but showed a significant effect against intracellular amastigotes of *L. amazonensis* and *L. infantum*, showing selectivity for the parasite when compared to the host cell. Furthermore, through transmission electron microscopy, it was possible to show that Marizomib induces extensive ultrastructural changes in amastigotes of *L. amazonensis* and *L. infantum*, such as the appearance of many vacuoles in the parasite cytoplasm and mitochondrial swelling. Marizomib was also shown to be effective in a murine model of cutaneous leishmaniasis, with a reduction in the size of lesions and parasite load in the footpads and draining lymph nodes of animals infected with *L. amazonensis*. It is also effective in a model of visceral leishmaniasis, with a reduction in parasite load in the spleen and liver of animals infected with *L. infantum*. Importantly, Marizomib, in the treatment regimens used, did not cause renal and hepatic acute toxicity to infected animals. These results highlight the antileishmanial potential of Marizomib, encouraging us to conduct preclinical tests in other animal models, as well as clinical trials.

## INTRODUCTION

Leishmaniases are diseases caused by protozoa of the *Leishmania* genus and are transmitted to humans and other mammals through the bites of sandflies, female insect vectors (1). There are two main clinical forms: cutaneous leishmaniasis and visceral leishmaniasis. The first is usually limited to a self-healing ulcer within 3–18 months, but it can also lead to scarring, disfigurement, and social stigmatization. Visceral leishmaniasis is the most severe form, systemic, and is often fatal if left untreated (2). Currently, more than 1 billion people live at risk of infection in endemic areas. It is estimated that 30,000 new cases of visceral leishmaniasis and more than 1 million new cases of cutaneous leishmaniasis occur annually (1).

One of the main strategies for controlling leishmaniasis is based on early diagnosis and treatment of infected patients. However, the current treatment of leishmaniasis is surrounded by a series of problems, such as many adverse effects, parenteral administration, long treatment time, and drug resistance (2, 3). Based on these limitations, the search for new drugs and other treatment regimens continues and is critically needed (3).

Marizomib is a β-lactone-γ-lactam irreversible proteasome inhibitor derived from the marine actinomycete *Salinispora tropica*, exhibiting remarkable specificity for the 20S proteasome (4) and has shown antitumor effects in Phase III clinical trials for the treatment of various types of glioblastoma (5). In *in vitro* and *in vivo* models of glioblastoma, Marizomib induces tumor cell death (6). Zhang and colleagues (7) found that Marizomib showed cytotoxic effects in cervical cancer cell lines, and its combination with Cisplatin resulted in more cytotoxicity and apoptosis against cervical cancer. Additionally, it was shown that Marizomib suppresses triple-negative breast cancer via proteasome and oxidative phosphorylation inhibition (8). Furthermore, in a preclinical study, it was demonstrated that Marizomib elicited a significant antitumor effect in a rodent intracranial model of malignant glioma (9).

Recently, a pre-clinical drug candidate, which has been shown to be a proteasome inhibitor, has been shown to have significant effects against relevant clinical isolates of *Leishmania donovani* and *L. infantum*. Furthermore, this compound showed promising *in vivo* effects and pharmacokinetic properties (10).

Due to these factors, in the context of leishmaniasis, the repositioning of Marizomib can also be an alternative. When compared to other proteasome inhibitors such as Bortezomib and Carfilzomib, Marizomib offers advantages such as bioavailability, enhanced stability, and the potential for a broader range of anti-cancer activity (5) due to its broader, faster-acting, and durable inhibition of all three 20S proteasome proteolytic activities in various models (4).

Based on the facts cited here, this work aimed to evaluate the *in vitro* effect of Marizomib on promastigotes and amastigotes of *L. amazonensis* and *L. infantum*, as well as to determine the effect of this proteasome inhibitor on *in vivo* models in mice infected with *L. amazonensis* and *L. infantum*.

## MATERIALS AND METHODS

### Compounds

Marizomib was purchased from Sigma-Aldrich (St. Louis, MO, USA) and dissolved in DMSO (Sigma-Aldrich) at a concentration of 10 mM. After dilution, it was stored at −20°C, according to the manufacturer’s instructions. Amphotericin B, obtained from Cristália, was used as a reference drug in antileishmanial tests. It was diluted in sterile deionized water.

### Parasites

In this work, we used two strains of *Leishmania amazonensis*: IFLA/BR/1967/PH8 (wild-type, WT) and IFLA/BR/1967/PH8 transfected with the gene of red fluorescent protein (RFP) and one strain of *L. infantum:* MHOM/MA/67/ITMAP-263. *L. amazonensis* WT and *L. infantum* were cultivated in BHI medium (Kasvi, São José dos Pinhais, PR, Brazil) supplemented with 10% fetal bovine serum (FBS) (Cultilab, Campinas, SP, Brazil), 0.1% penicillin and streptomycin solution (Sigma-Aldrich, St. Louis, MO, USA), 0.5 mg/mL folic acid (Sigma-Aldrich), and 5 mg/mL hemin (Sigma-Aldrich). *L. amazonensis* RFP was grown in 199 medium (HiMedia Laboratories Pvt. Ltd., Mumbai, India) supplemented with 10% FBS, 0.1% penicillin and streptomycin solution, 0.5 mg/mL folic acid, 5 mg/mL hemin, 4 mg/mL adenine (Sigma-Aldrich), 0.2 mg/mL D-biotin (Sigma-Aldrich), and MEM vitamin solution (Thermo Fisher Sci., Waltham, MA, USA). Parasites were grown at 25°C and were constantly isolated from lesions of BALB/c mice.

### Mice

Female BALB/c mice were obtained from the Central Animal Facility of Universidade Federal do Rio de Janeiro (UFRJ) and Universidade Federal de Juiz de Fora (UFJF). All procedures for the use and maintenance were performed according to the protocols approved by the Ethical Committee for Animal Handling (CEUA 042/2019 from Fiocruz, 080/2018 from UFRJ, and 001/2023 from UFJF). The mice were kept at 22°C and under controlled light/dark cycle conditions, with access to water and food *ad libitum*. All the animals used were 6- to 10-week-old and weighed 20–25 g.

### *In vitro* cytotoxicity assay against peritoneal macrophages

To determine the toxicity of Marizomib on mammalian cells, peritoneal macrophages from BALB/c mice were used. These cells were obtained by peritoneal lavage and distributed in 96-well plates at 2 × 10^6^ cells/mL in RPMI medium (Cultilab) containing 10% FBS and a 0.5% penicillin and streptomycin solution (complete RPMI) in a final volume of 200 µL/well. The plates were incubated for 1 h at 37°C and 5% CO_2_, washed with phosphate-buffered saline (PBS), and then re-incubated in complete RPMI medium overnight at 37°C with 5% CO_2_. Marizomib was added at different concentrations (1,000, 100, 10, 1, 0.1, and 0.01 nM) and incubated for 72 h at 37°C and 5% CO_2_. Controls that did not receive any treatment were included. Cell viability was assessed by incubating the cells with MTT (Sigma-Aldrich) for 4 h, followed by the addition of isopropanol-HCl to stop the reaction. The absorbances were read using a spectrophotometer (Multiskan EX) at 570 nm. Three independent experiments in triplicate were performed.

### *In vitro* anti-promastigote assay

Promastigote forms of *L. amazonensis* (WT) or *L. infantum*, in the log phase of growth, were distributed in 96-well plates at 2 × 10^6^ or 3 × 10^6^ promastigotes/mL, respectively. Marizomib was added at different concentrations (1,000, 100, 10, 1, 0.1, and 0.01 nM) and incubated with parasites for 72 h at 25°C. After this time, MTT was added and incubated for 4 h. As previously described, the reaction was stopped by adding an isopropanol/HCl solution, and the reading was performed at 570 nm using a spectrophotometer (Multiskan EX). Some wells did not receive treatment (control), while others were treated with amphotericin B (positive control). The assay was performed in triplicate and repeated three times.

### *In vitro* anti-amastigote assay

Peritoneal macrophages from BALB/c mice were obtained by peritoneal lavage and distributed in 24-well plates at 2 × 10^6^ cells/mL in RPMI medium (Cultilab) containing 10% FBS and a 0.5% penicillin and streptomycin solution (complete RPMI). The plates were incubated for 1 h at 37°C and 5% CO_2_, washed with phosphate-buffered saline, and then re-incubated in complete RPMI medium overnight. The cells were washed again with PBS and infected with stationary growth phase *L. amazonensis* (RFP) promastigotes at a 10:1 ratio for 4 h at 33°C or with *L. infantum* promastigotes at a 10:1 ratio for 24 h at 37°C. After each incubation period, the plates were washed with PBS, Marizomib was added at different concentrations (1,000, 100, 10, 1, 0.1, and 0.01 nM) and incubated for 72 h. For the experiment with *L. infantum*, FBS was replaced with 4% horse serum (Laborclin). For *L. amazonensis*–RFP, after 72 h, each well was scraped to remove attached cells. The reading was performed using a spectrofluorimeter (FLx800, BioTek Instruments, Winooski, VT, USA) at 530 and 590 nm of excitation and emission, respectively. For *L. infantum*, the coverslips pre-added into the wells of 24-well plates were removed, the cells were fixed with ethanol, stained with Giemsa, and dehydrated in an acetone/xylol series. The coverslips were placed on glass slides, and for each coverslip, a total of 100 macrophages were assessed for the count of intracellular parasites. For both experiments, some wells did not receive treatment (control), and others were treated with amphotericin B (positive control). All assays were performed in triplicate and repeated three times.

### Calculation of the selectivity index

The selectivity index (SI) was calculated manually by dividing the CC_50_ value in macrophages by the IC_50_ value in amastigotes of *L. amazonensis* or *L. infantum*.

### Transmission electron microscopy of *L. amazonensis* and *L. infantum* amastigotes

Peritoneal macrophages already infected with *L. amazonensis* were treated with 15, 30, and 60 nM of Marizomib for 24 h. In parallel, peritoneal macrophages already infected with *L. infantum* were treated with 10, 20, and 40 µM of Marizomib for 24 h. These concentrations correspond to approximately 1/2×, 1×, and 2× the IC_50_ value of this compound in *L. amazonensis* or *L. infantum* amastigotes. After treatment, the cells were washed with PBS, fixed in 2.5% glutaraldehyde in 0.1 M sodium cacodylate buffer (at room temperature for 1 h), and post-fixed in 1% osmium tetroxide and 0.8% potassium ferrocyanide solution. An increasing concentration series of acetone (70%, 90%, and 100%) was used to dehydrate the cells, which were next embedded in Epon resin and polymerized at 60°C. Ultrathin sections (60–70 nm thick) were stained with 5% uranyl acetate and 3% lead citrate and examined using a Jeol 358 JEM 1011 (Tokyo, Japan) transmission electron microscope. Untreated and DMSO-treated controls were included.

### *In vivo* assay against *L. amazonensis*

Female BALB/c mice were subcutaneously infected in the right hind footpad with 2 × 10^6^ stationary phase *L. amazonensis* promastigotes (PH8–WT) in PBS. The course of the infection was monitored by measuring footpad thickness with a dial caliper. The treatment started 10 days after infection and was performed by intraperitoneal route, two to three times a week, in a total of eight doses of 0.15 mg Marizomib/kg of body weight or 5 mg amphotericin B/kg of body weight. The animals received treatment on days 10, 12, 14, 18, 20, 24, 27, and 30. In the control group, the animals received a vehicle (DMSO 5% in PBS). Three days after the end of treatment, the animals were anesthetized, blood was collected by cardiac puncture, and euthanasia was performed by cervical dislocation, followed by perforation of the diaphragm. The infected footpads were excised and placed for 1 min in 70% alcohol for disinfection. The popliteal lymph node and spleen were also removed. Footpads, lymph nodes, and spleens were macerated. A pre-dilution of 1:500 was prepared for the footpads. In 96-well plates, 50 µL of the homogenates (for spleens and lymph nodes) or 50 µL of the pre-dilution (for footpads) were serially diluted fourfold in 150 µL of BHI medium per well. The plates were incubated at 26°C for 7 days. At the end of this period, the plates were visually evaluated under an optical microscope, and the last well in which parasites could be seen was considered in determining the parasite load. Thus, determining the parasite load/g was as follows: 4^*N*^ × *Y*/*Z*, where *N* = number of last well in which parasites could be seen; *Y* = pre-dilution and *Z* = organ weight.

The acute toxicity of Marizomib was also determined. AST, ALT, and creatinine levels were measured in the serum of animals infected with *L. amazonensis* and treated with Marizomib (0.15 mg/kg) and compared with levels in the serum of control animals, which received PBS containing 5% DMSO. At the end of the treatment and before euthanasia, blood was collected, and the serum was separated by centrifuging the whole blood at 5,000 *g* for 5 min. AST, ALT, and creatinine levels were determined using kinetic detection kits obtained from Bioclin.

### *In vivo* assay against *L. infantum*

For the visceral murine model, female BALB/c mice were intraperitoneally infected with 2.5 × 10^7^ stationary-phase *L. infantum* promastigotes. Ten days after infection, the treatment was initiated and performed intraperitoneally with five doses of 0.15 mg Marizomib/kg of body weight or 5 mg amphotericin B/kg of body weight, and the control group received a vehicle (DMSO 5% in PBS). The animals received treatment on days 10, 13, 16, 19, and 22. Three days after the end of treatment, the animals were anesthetized, blood was collected by cardiac puncture, and euthanasia was performed by cervical dislocation, followed by perforation of the diaphragm. Liver and spleen were also removed and macerated. In 96-well plates, 200 µL of the homogenates was serially diluted in BHI medium. The plates were incubated at 26°C for 7 days. At the end of this period, the plates were visually evaluated under an optical microscope, and the last well in which parasites could be seen was considered in the determination of the parasite load. To calculate the parasite load, the formula already mentioned in the previous section was used.

### Statistical analyses

Statistical analyses were performed using GraphPad Prism 8 software. Statistical differences between mean values were evaluated by applying an unpaired *t*-test. The IC_50_ and CC_50_ were obtained by nonlinear regressions, also using GraphPad Prism 8.

## RESULTS

### Marizomib had a significant effect on intracellular amastigotes of *L. amazonensis* and *L. infantum*

**Fig 1:**
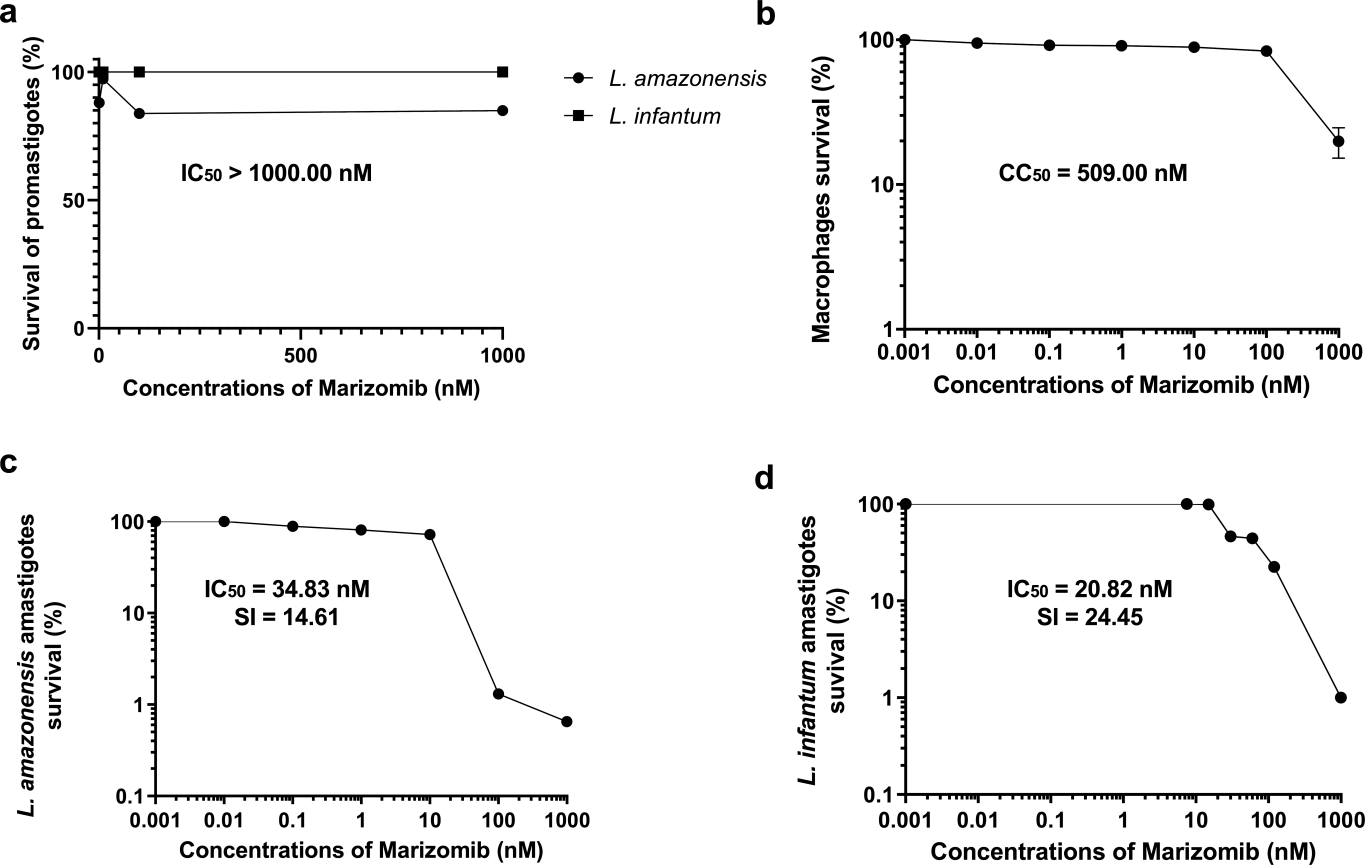
Effects of Marizomib in peritoneal macrophages and *Leishmania* spp. Promastigotes, peritoneal macrophages, or amastigotes were treated with different concentrations of Marizomib for 72 h. (**a**) Antipromastigote effect of Marizomib, determined by the MTT colorimetric method, with subsequent calculation of the IC_50_ value. (**b**) Marizomib toxicity in peritoneal macrophages of BALB/c mice, also assessed by the MTT colorimetric method, with subsequent calculation of the CC_50_ value. (**c**) *Leishmania amazonensis* antiamastigote effect of Marizomib, determined by reading the fluorescence intensity of parasites transfected with RFP, with calculation of the IC_50_ value and selectivity index, given by dividing the value of CC_50_ by IC_50_. (**d**) *Leishmania infantum* antiamastigote effect of Marizomib, assessed by counting intracellular parasites after staining with Giemsa, with calculation of the IC_50_ value and SI.

Initially, we determined the effect of Marizomib on promastigote forms of *L. amazonensis* and *L. infantum*. Figure 1a shows that Marizomib was not effective against *L. amazonensis* and *L. infantum* promastigotes up to the maximum concentration used, which was 1,000 nM. Subsequently, we determined the toxicity of Marizomib in mammalian cells, and it was observed that this compound presented considerable toxicity, with a CC_50_ value of 509.00 ± 54.6 nM (Fig. 1b). However, Marizomib proved to be effective against intracellular amastigote forms of *L. amazonensis* (IC_50_ = 34.83 ± 2.6 nM) and *L. infantum* (IC_50_ = 20.82 ± 5.5 nM) (Fig. 1c and d). Calculating the selectivity index, it can be seen that Marizomib was approximately 15 times more toxic to *L. amazonensis* amastigotes (SI = 14.61) and approximately 25 times more toxic to *L. infantum* amastigotes (SI = 24.45) compared to peritoneal macrophages (Fig. 1c and d). These results highlight the antileishmanial potential of Marizomib.

Amphotericin B was used as a reference drug and presented IC_50_ values for promastigotes of *L. amazonensis* = 0.10 µM and *L. infantum* = 0.12 µM. For amastigotes, amphotericin B showed IC_50_ values of 0.18 and 0.05 µM in *L. amazonensis* and *L. infantum*, respectively.

### Marizomib caused several ultrastructural changes in amastigotes of *L. amazonensis* and *L. infantum*

**Fig 2:**
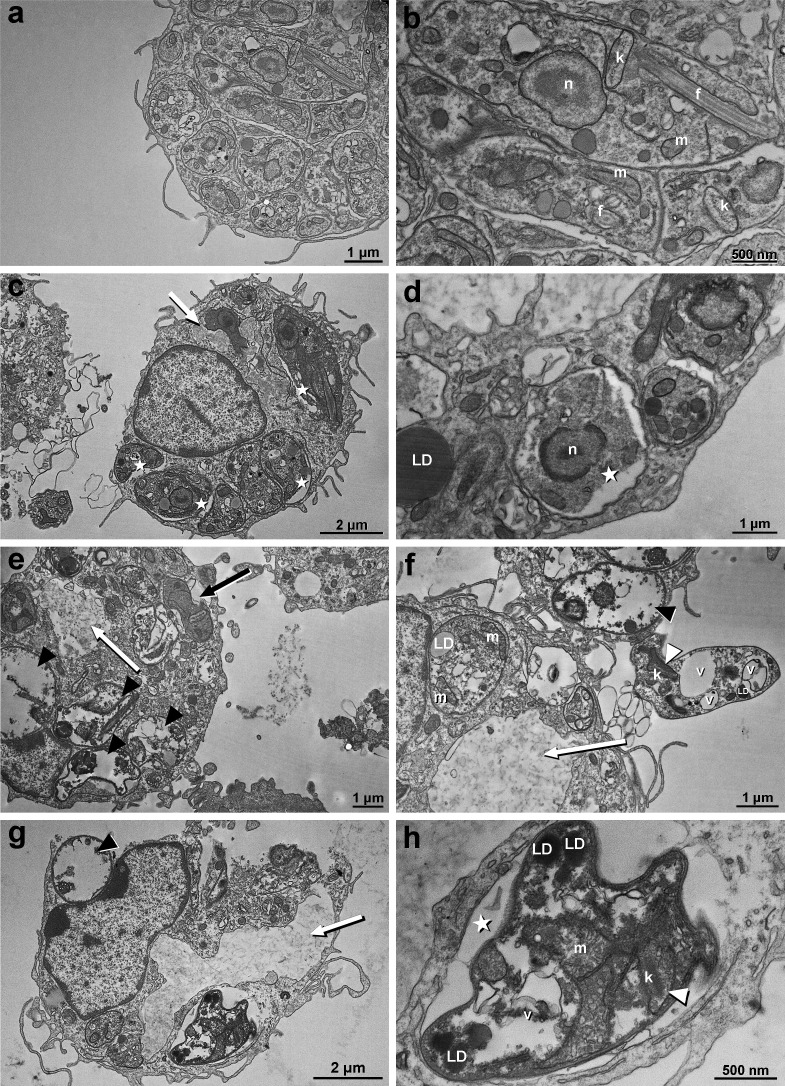
Ultrastructural analysis of the effects of Marizomib on *Leishmania amazonensis* amastigotes. Peritoneal macrophages were infected with *L. amazonensis* and treated with different concentrations of Marizomib. The cells were then prepared for transmission electron microscopy. (**a and b**) Control, demonstrating the integrity of intracellular parasites. (**c and d**) Treatment with Marizomib at 15 nM: presence of amorphous material in some regions of the parasitophorous vacuole (white arrow); a decrease in the space occupied by the parasite within the parasitophorous vacuole (star) was also observed. (**e and f**) Treatment with Marizomib at 30 nM: greater damage to the parasites is observed. Large vacuoles (V) were seen in the parasite cytoplasm, and the appearance of membrane profiles surrounding the parasitophorous vacuole (black arrow) was observed. In panel f, mitochondrial swelling was observed (white arrowhead). (**g and h**) Treatment with Marizomib at 60 nM: parasites are completely altered. Many parasites, after treatment, exhibited severe cytoplasmic alterations, with cytoplasmic content extravasation. In some cases, only a small amount of cytoplasmic material (such as glycogen, structures, and organelles) remained, which is characteristic of cell death (black arrowhead) (e). It was possible to note the presence of large vacuoles with membrane profiles, vesicles, and LD inside them (**h**). Mitochondrial swelling is also observed (white arrowhead), as well as a decrease in the volume occupied by the parasite inside the parasitophorous vacuole (star). Panel h is a higher magnification of the same cell as the one shown in panel g. N, nucleus; m, mitochondria; f, flagellum; and k, kinetoplast.

Figure 2 shows that treatment of *L. amazonensis* intracellular amastigotes with Marizomib caused numerous ultrastructural changes in the parasites. At a concentration of 15 nM Marizomib, the presence of amorphous material was observed in some regions of the parasitophorous vacuole (white arrow) (Fig. 2c). In addition, a decrease in the space occupied by the parasite within the parasitophorous vacuole (star) was also observed (Fig. 2c and d). Parasites treated with Marizomib at 30 nM were even more altered. The appearance of membrane profiles surrounding the parasitophorous vacuole (black arrow) and the presence of amorphous material in the macrophage (white arrow) were observed (Fig. 2e). Additionally, after treatment, many parasites exhibited severe cytoplasmic alterations, with cytoplasmic content extravasation. In some cases, only a small amount of cytoplasmic material (such as glycogen, structures, and organelles) remained, which is characteristic of cell death (black arrowhead) (Fig. 2e). Many vacuoles were observed in the parasite cytoplasm, in addition to the presence of amorphous material on macrophages (white arrow), dead parasites (black arrowhead), and mitochondrial swelling (white arrowhead) (Fig. 2f). After treatment with 60 nM of Marizomib, we observed dead parasites (black arrowhead) and the presence of amorphous material in the parasitophorous vacuole (white arrow) (Fig. 2g). The presence of large vacuoles with membrane profiles and internal lipid droplets (LD) (Fig. 2h) was also noted. Mitochondrial swelling was also observed (white arrowhead), as well as a decrease in the volume occupied by the parasite within the parasitophorous vacuole (star) (Fig. 2h).

**Fig 3:**
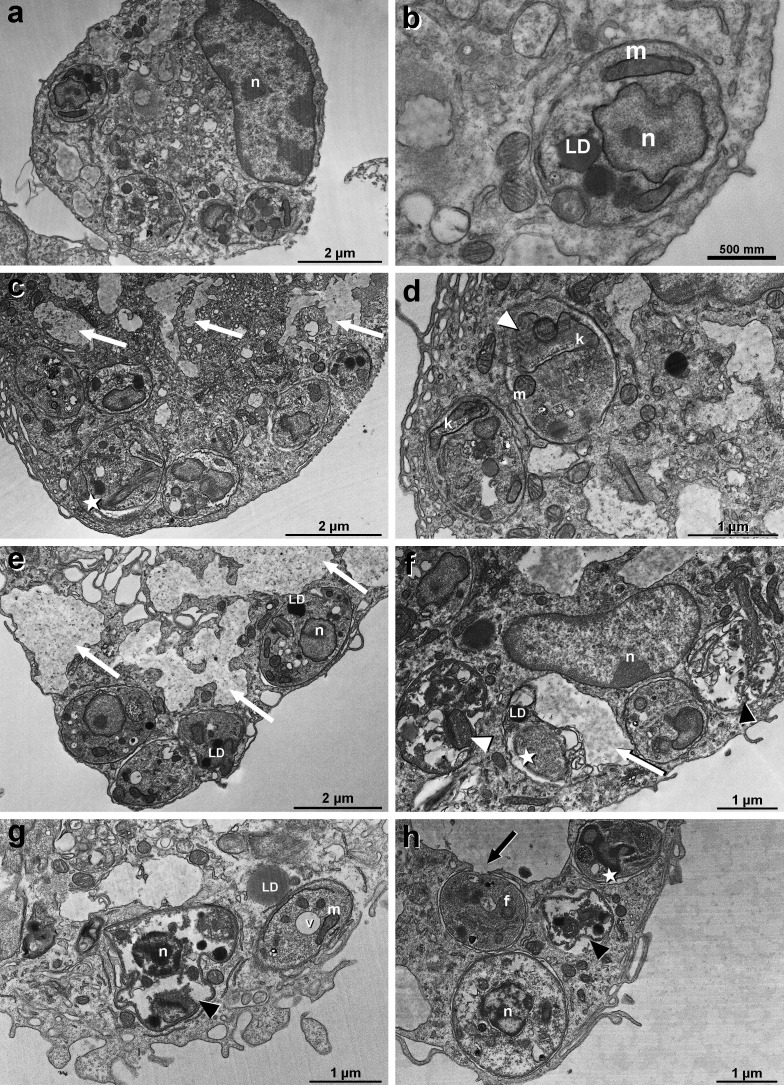
Ultrastructural analysis of the effects of Marizomib on *Leishmania infantum* amastigotes. Peritoneal macrophages were infected with *L. infantum* and treated with different concentrations of Marizomib. The cells were then prepared for transmission electron microscopy. (**a and b**) Control, demonstrating the integrity of intracellular parasites. (**c and d**) Treatment with Marizomib at 10 nM: presence of vacuoles with amorphous content in macrophages (white arrow), a decrease in the volume occupied by parasites in the parasitophorous vacuole (star), and swelling of the mitochondria (white arrowhead) were observed. (**e and f**) Treatment with Marizomib at 20 nM: increase in vacuoles with amorphous content (white arrows) and more damaged parasites, with loss of a large part of the cytoplasmic material (black arrowhead). (**g and h**) Treatment with Marizomib at 40 nM: several stages of changes in different parasites, such as decrease in the volume occupied by the parasites in the parasitophorous vacuole (star), very damaged parasites with loss of a large part of the cytoplasmic material (black arrowhead), and the presence of a rupture/opening (black arrow) in the macrophage vacuole membrane next to *L. infantum* (**h**). N, nucleus; m, mitochondria; f, flagellum; and K, kinetoplast.

In *L. infantum* intracellular amastigotes, the treatment with Marizomib also caused numerous ultrastructural changes (Fig. 3). Marizomib at 10 nM induced the appearance of vacuoles with amorphous content in macrophages (white arrow), decrease in the volume occupied by parasites in the parasitophorous vacuole (star), and swelling of the mitochondria (white arrowhead) (Fig. 3c and d). Treatment with Marizomib at 20 nM also induced the appearance of vacuoles with amorphous material in host cells (white arrow), decrease in the volume occupied by parasites inside parasitophorous vacuole (star), and swelling of the mitochondria (white arrowhead) (Fig. 3e and f). Additionally, we observed parasites with loss of a large part of the cytoplasmic material (black arrowhead) (Fig. 3f). After treatment with Marizomib at 40 nM, we observed the presence of very damaged parasites with loss of a large part of the cytoplasmic material (black arrowhead), decrease in the volume occupied by the parasites in the parasitophorous vacuole (star), appearance of a vacuole in the cytoplasm of amastigotes, and presence of rupture/opening (black arrow) of the macrophage vacuole membrane (Fig. 3g and h). These results demonstrated the direct effect of Marizomib against intracellular amastigotes.

### Marizomib showed a significant effect against *L. amazonensis in vivo*, without showing toxicity

**Fig 4:**
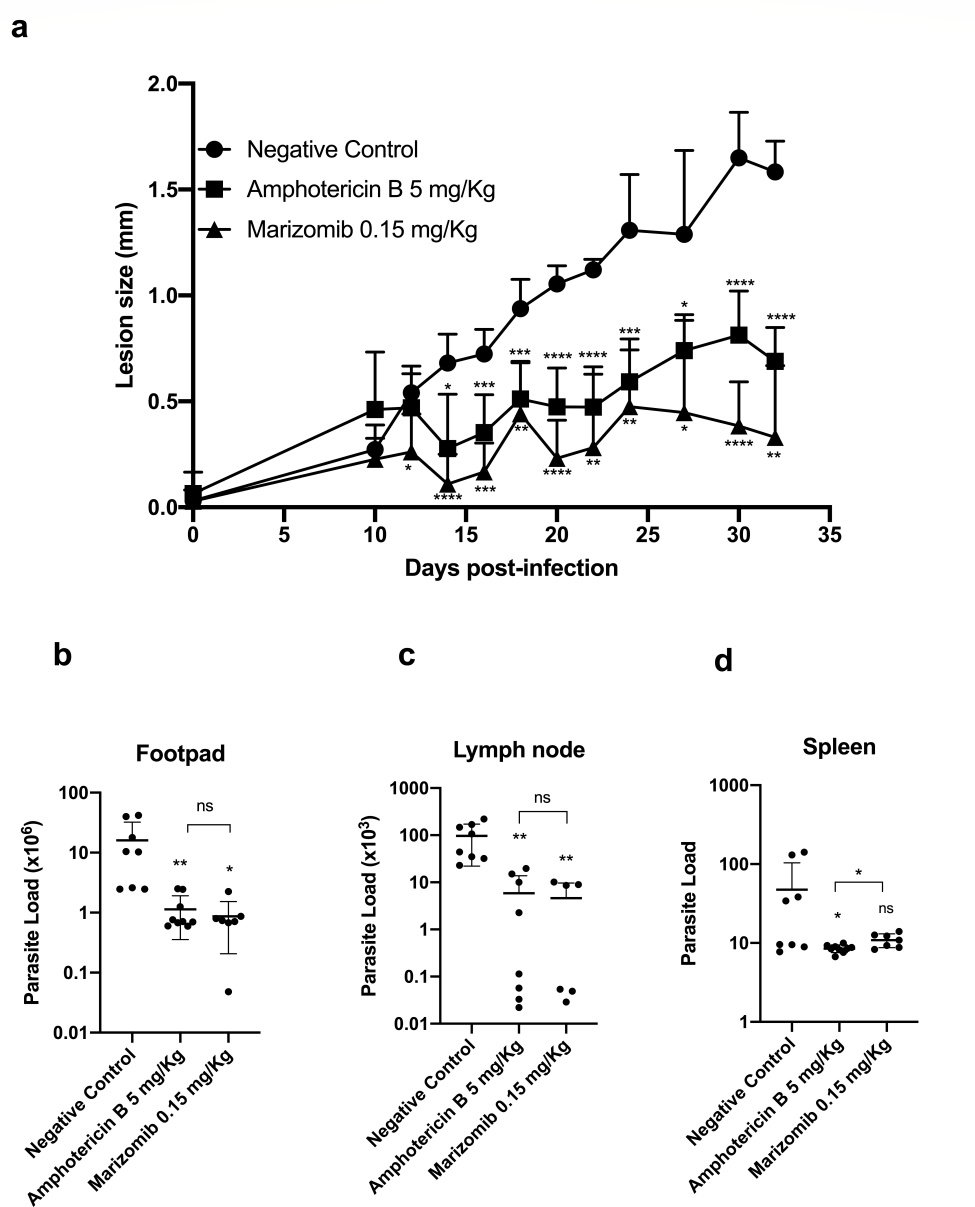
*In vivo* effect of Marizomib in BALB/c mice infected with *L. amazonensis*. BALB/c mice were subcutaneously infected in the right hind footpad with *L. amazonensis* promastigotes (PH8–WT) in PBS. The treatment started 10 days after infection and was performed by intraperitoneal route, two to three times a week, in a total of eight doses of 0.15 mg Marizomib/kg of body weight or 5 mg amphotericin B/kg of body weight. The animals received treatment on days 10, 12, 14, 18, 20, 24, 27, and 30. In the control group, the animals received a vehicle (DMSO 5% in PBS). The course of the infection was monitored by measuring footpad thickness with a dial caliper. Three days after the end of treatment, the animals were anesthetized, and infected footpads, popliteal lymph nodes, and spleen were also removed and macerated to determine the parasite load. (**a**) Measurement of lesion sizes. Only the upper part of the deviations was shown to facilitate the visualization of the graph. (**b**) Parasite load in infected footpads. (**c**) Parasite load in draining lymph nodes. (**d**) Parasite load in the spleen.

Treatment of BALB/c mice infected with *L. amazonensis* with Marizomib caused a significant reduction in the size of footpad lesions from the 12th day (second treatment) until the last day (32nd) (Fig. 4a). Remarkably, Marizomib was able to cause, in general, a greater reduction in the size of the lesions than amphotericin B, which was used as the reference drug (Fig. 4a). Additionally, treatment with Marizomib significantly reduced the parasite load in the footpads and popliteal lymph nodes of animals infected with *L. amazonensis*, when compared to the negative control, which received DMSO 5% in PBS (Fig. 4b and c). Marizomib was not able to reduce the parasite load in the spleen of the animals; however, there was a strong tendency toward this (Fig. 4d). Treatment with amphotericin B, used as a reference drug, reduced the parasite load in the footpads, lymph nodes, and spleen of infected animals (Fig. 4b through d). Importantly, the effect of Marizomib in reducing parasite loads in the footpads and lymph nodes was statistically similar to the effect of the reference drug, amphotericin B (Fig. 4b and c).

**Fig 5:**
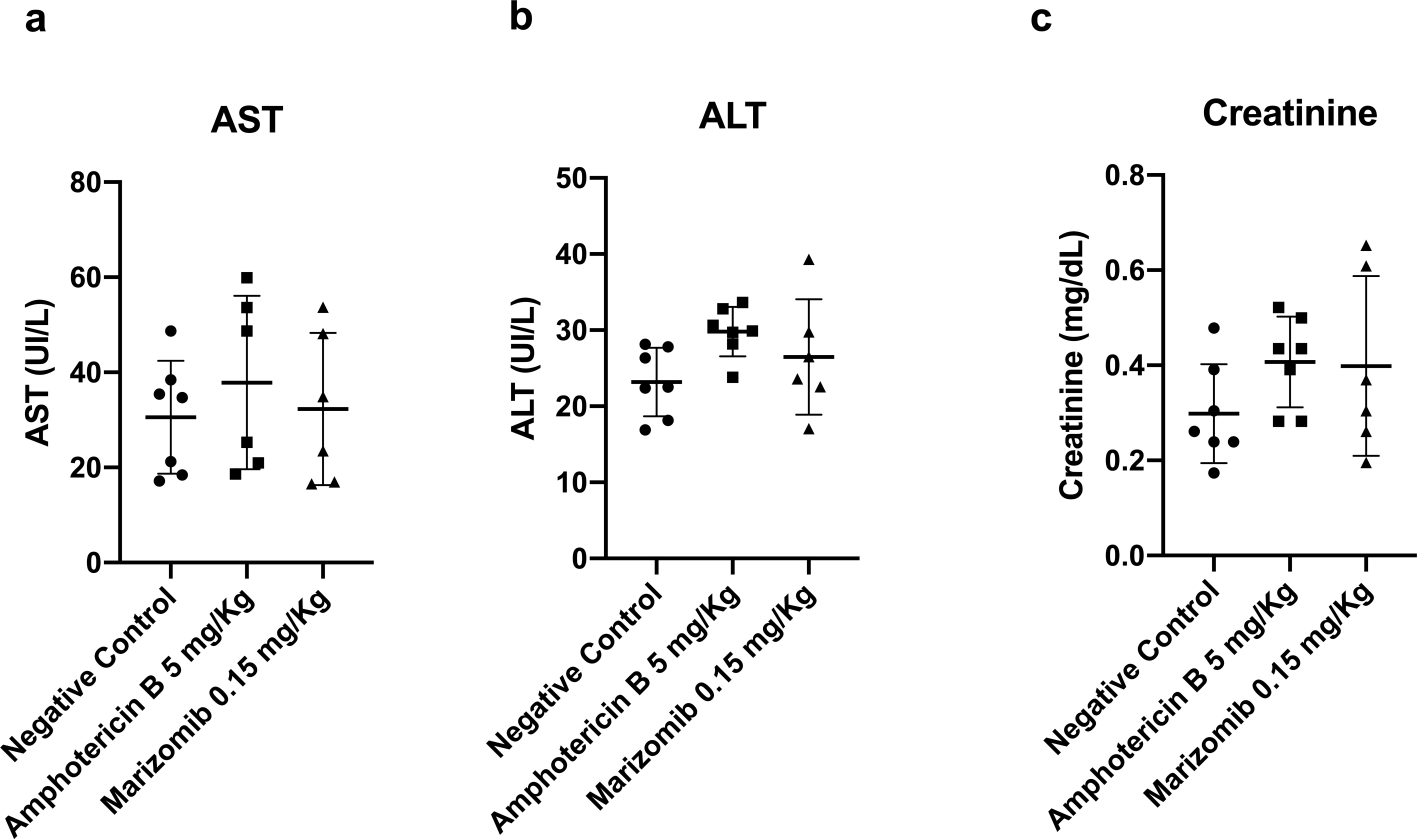
*In vivo* toxicity of Marizomib in BALB/c mice infected with *L. amazonensis*. AST (**a**), ALT (**b**), and creatinine (**c**) levels were determined in the serum of animals infected with *L. amazonensis* and treated with Marizomib and were compared with those of control animals, which received PBS containing 5% DMSO. At the end of the treatment and before euthanasia, blood was collected by cardiac puncture, and the serum was analyzed. AST, ALT, and creatinine levels were determined using kinetic detection kits obtained from Bioclin.

It is important to highlight that treatment with Marizomib did not change the levels of AST, ALT, and creatinine in the serum of animals infected with *L. amazonensis* when compared to the negative control (Fig. 5a through c). These data show that Marizomib did not cause acute renal and hepatic toxicity in the animals.

### Marizomib was significantly effective against *L. infantum in vivo*

**Fig 6:**
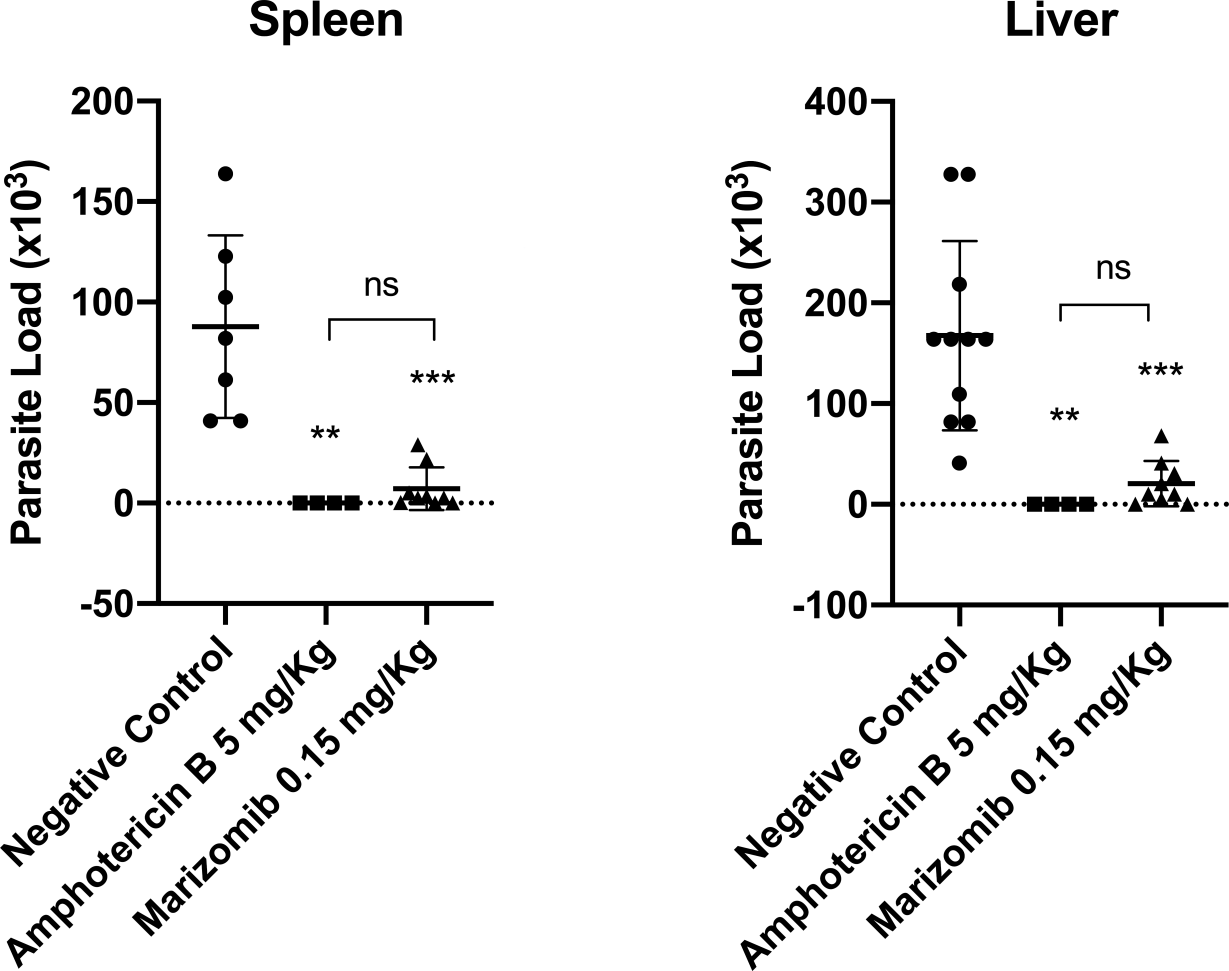
*In vivo* effect of Marizomib in BALB/c mice infected with *L. infantum*. BALB/c mice were intraperitoneally infected with *L. infantum* promastigotes. Ten days after infection, the treatment was initiated and performed intraperitoneally with five doses of 0.15 mg Marizomib/kg of body weight or 5 mg amphotericin B/kg of body weight, and the control group received a vehicle (DMSO 5% in PBS). The animals received treatment on days 10, 13, 16, 19, and 22. Three days after the end of treatment, the animals were anesthetized, the liver and spleen were removed and macerated for the determination of the parasite load.

We also evaluated the effect of Marizomib in a visceral leishmaniasis model using BALB/c mice infected with *L. infantum*. As was observed with *L. amazonensis*, treatment of mice with five doses of Marizomib resulted in a significant reduction in the parasite load in the liver and spleen of infected animals (Fig. 6). Amphotericin B was used as a reference drug and also significantly reduced the parasite load in the liver and spleen of animals infected with *L. infantum* (Fig. 6). Importantly, the effect of Marizomib in reducing parasite loads in the liver and spleen was statistically similar to the effect of the reference drug, amphotericin B (Fig. 6).

Additionally, treatment with Marizomib did not cause renal and hepatic acute toxicity in animals infected with *L. infantum*, as there was no change in AST, ALT, and creatinine levels (data not shown).

## DISCUSSION

Although leishmaniasis is an ancient disease, its treatment is still a challenge due to various problems such as toxicity, adverse effects, long duration of treatment, high cost, and parasite resistance. To try to overcome these limitations, the research for new medicines and other treatment alternatives continues (3). In *Leishmania* spp., proteases are involved in several biological processes of this parasite (11). Our research group recently demonstrated that inhibition of serine proteases of *L. amazonensis* promastigotes, through treatment with different inhibitors, reduces the survival of parasites within macrophages during the first 72 h of infection (12). Due to these factors, protease inhibitors have been tested in *Leishmania*, with the aim of discovering new treatment alternatives (13, 14).

Proteasomes are large, multicatalytic protein complexes that degrade many cellular proteins into peptides, contributing to homeostasis and proper cell functioning (15). The ubiquitin/proteasome pathway is responsible for the degradation of approximately 80% of intracellular proteins, and this is a highly regulated process (15). Ubiquitinated proteins are recognized by regulatory subunits on the 26S proteasome complex, the ubiquitin chains are removed and recycled, and the protein is unfolded and translocated into the interior by the proteasome where it is cleaved into peptides by the active protease subunits (15). Proteasome inhibitors are a new class of drugs for the treatment of multiple myeloma and some types of lymphoma. Currently, there are many proteasome inhibitors in clinical trials for different types of cancer (15). Bortezomib was the first proteasome inhibitor to be approved by the Food and Drug Administration (FDA) in 2003, followed by Carfilzomib in 2012 and Ixazomib in 2015 (15). The *L. mexicana* 20S proteasome was purified and characterized by Robertson (16) in 1999, which also showed, using proteasome inhibitors, that this protease is essential for parasite growth *in vitro*. The effects of proteasome inhibitors in *L. mexicana* are in part caused by cell cycle arrest due to inhibition of the destruction of cell cycle regulators (16). A pre-clinical drug candidate, which acts through proteasome inhibition, selectively inhibiting the parasite enzyme when compared to the host cell, was shown to have significant effects against relevant clinical isolates of *L. donovani* and *L. infantum* and showed promising *in vivo* effects and pharmacokinetic properties (10).

Marizomib is a proteasome inhibitor that, used alone or in combination with other drugs, has shown significant antitumor effects (6–9). In the present work, we evaluated the effect of Marizomib on promastigotes and amastigotes of *L. amazonensis* and *L. infantum*. Interestingly, Marizomib showed no effect on promastigotes of both *Leishmania* species (IC_50_ > 1,000.00 nM). However, it was significantly effective on intracellular amastigotes of *L. amazonensis* (IC_50_ = 34.83 nM = 0.03483 µM) and *L. infantum* (IC_50_ = 20.82 nM = 0.0282 µM), presenting selectivity for the parasite (SI = 14.61 and 24.45 for *L. amazonensis* and *L. infantum*, respectively) when compared to the host cell. These results show that despite presenting toxicity to host cells, Marizomib is approximately 15 times more active against *L. amazonensis* amastigotes and approximately 25 times more toxic to *L. infantum* amastigotes, when compared to its effect on peritoneal macrophages.

It has been previously reported that amastigotes of *L. mexicana* contain high protease activity, about 20 times higher than in promastigote forms, which may be an important primary factor for the survival and growth of amastigotes within the parasitophorous vacuole (17). However, the literature is unclear regarding proteasome activity between the two evolutionary stages of *Leishmania* spp. Here, we show that amastigotes of *L. amazonensis* and *L. infantum* are sensitive to Marizomib, which was not the case with promastigotes. However, most studies only show the effect of proteasome inhibitors on amastigotes, which is the evolutionary stage present in humans (10, 18). At this point, it is important to emphasize that considering the treatment strategy for leishmaniasis in humans, Marizomib fulfills this role excellently, as it has an effect precisely on the evolutionary form of the parasite found in humans and other mammals, which is the intracellular amastigote.

In the work of Wyllie and colleagues (10), the best proteasome inhibitor (compound 8) from a series of compounds showed an excellent effect against intracellular amastigotes of *L. donovani*, with an IC_50_ value of 1.6 µM. This concentration is much higher than that of Marizomib in the present work. Furthermore, in the work by Nagle and colleagues (18), a series of proteasome inhibitors were tested, which showed excellent effectiveness, with the two most effective in *L. donovani* amastigotes: LXE408 (IC_50_ = 0.04 µM) and GNF6702 (IC_50_ = 0.02 µM). The concentration is similar to the effect of Marizomib demonstrated here.

We also evaluated the ultrastructural changes caused by Marizomib in intracellular amastigote forms of *L. amazonsensis* and *L. infantum*. Many changes were observed in the parasites, highlighting the reduction in body volume, the appearance of cytoplasmic vacuoles, and mitochondrial swelling. These ultrastructural changes induced by Marizomib are likely contributing to parasite death after treatment. The induction of mitochondrial alterations is always important in these parasites, as this organelle is unique in *Leishmania* spp. and has significant differences compared to mammalian mitochondria. Interestingly, Wyllie and colleagues (10) also observed the appearance of intracytoplasmic vesicles in *L. donovani* promastigotes treated with a proteasome inhibitor (compound 7). In addition, treatment with compound 7 resulted in an accumulation of cells in the G2/M phase and a decrease in the proportion of cells in G1 and S phases, events compatible with what had been previously reported by Robertson (16) and related to inhibition of destruction of cell cycle regulators, which helps to reduce the growth of the parasite *in vitro* (10). In tumor cells, Zhang and colleagues (7) found that Marizomib induced apoptosis against cervical cancer. In cancer cells, the target of proteasome inhibitors appears to be the chymotrypsin-like β5 catalytic subunit. However, it is possible that many pathways contribute to this antitumor mechanism, with many key proteins being involved, such as NF-κB, p53, and cyclin. Proteasome inhibitors cause the accumulation of unfolded and misfolded proteins, which triggers the unfolded protein response and consequently apoptosis (15). The extrinsic pathway, via caspase-8, and the intrinsic pathway, via caspase-9, have also been proposed to be involved (15).

The *in vivo* effect of Marizomib in a murine model of cutaneous and visceral leishmaniasis was also evaluated using BALB/c mice infected with *L. amazonensis* or *L. infantum*. Marizomib administered intraperitoneally in eight doses reduced lesion size and parasite load in the footpads and lymph nodes in animals infected with *L. amazonensis*. When administered intraperitoneally in five doses, it reduced parasite load in the liver and spleen of animals infected with *L. infantum*. Overall, the effect of Marizomib was statistically similar to the effect of the reference drug, amphotericin B. In both treatment regimens, Marizomib did not cause acute hepatic or renal toxicity to infected animals, as there was no change in AST, ALT, and creatinine levels. These results highlight the antileishmanial potential of Marizomib in preclinical tests, using BALB/c mice infected with *L. amazonensis* or *L. infantum*. This compound was shown to be effective in models of cutaneous and visceral leishmaniasis.

The proteasome inhibitor LXE408, administered orally at 1, 3, and 10 mg/kg of body weight, also significantly reduced the parasite load in the liver of animals infected with *L. donovani* in a similar or even better way when compared to miltefosine (18). In addition, compound 8, another proteasome inhibitor, administered orally at 25 mg/kg of body weight, reduced the parasite load in the liver of animals infected with *L. donovani,* similar to miltefosine (10). In our study, using 0.15 mg Marizomib/kg of body weight, we had a similar effect that was observed before with LXE408 and compound 8, indicating the use of Marizomib as an alternative for leishmaniasis treatment.

It is important to highlight that Marizomib is used in combination with other antitumor drugs for the treatment of different types of cancer, obtaining interesting results, which may also be an alternative for the treatment of leishmaniasis. Associating Marizomib with other antileishmanial drugs, such as amphotericin B and miltefosine, is a strategy that we intend to carry out as a future perspective.

In conclusion, this work highlights the *in vitro* and *in vivo* antileishmanial potential of Marizomib against *L. amazonensis* and *L. infantum*, opening perspectives for preclinical tests in other animal models and clinical tests, as well as for the development of new, more effective molecules.
